# Neuromelanin‐Sensitive Magnetic Resonance Imaging Using DANTE Pulse

**DOI:** 10.1002/mds.28417

**Published:** 2020-12-14

**Authors:** Sonoko Oshima, Yasutaka Fushimi, Tomohisa Okada, Satoshi Nakajima, Yusuke Yokota, Atsushi Shima, John Grinstead, Sinyeob Ahn, Nobukatsu Sawamoto, Ryosuke Takahashi, Yuji Nakamoto

**Affiliations:** ^1^ Department of Diagnostic Imaging and Nuclear Medicine Graduate School of Medicine, Kyoto University Kyoto Japan; ^2^ Human Brain Research Center Graduate School of Medicine, Kyoto University Kyoto Japan; ^3^ Department of Human Health Sciences Graduate School of Medicine, Kyoto University Kyoto Japan; ^4^ Siemens Healthineers Portland Oregon USA; ^5^ Siemens Healthineers San Francisco California USA; ^6^ Department of Neurology Graduate School of Medicine, Kyoto University Kyoto Japan

**Keywords:** Parkinson's disease, magnetic resonance imaging, neuromelanin, substantia nigra

## Abstract

**Background:**

Neuromelanin‐sensitive magnetic resonance imaging techniques have been developed but currently require relatively long scan times.

The aim of this study was to assess the ability of black‐blood delay alternating with nutation for tailored excitation‐prepared T1‐weighted variable flip angle turbo spin echo (DANTE T1‐SPACE), which provides relatively high resolution with a short scan time, to visualize neuromelanin in the substantia nigra pars compacta (SNpc).

**Methods:**

Participants comprised 49 healthy controls and 25 patients with Parkinson's disease (PD). Contrast ratios of SNpc and hyperintense SNpc areas, which show pixels brighter than thresholds, were assessed between DANTE T1‐SPACE and T1‐SPACE in healthy controls. To evaluate the diagnostic ability of DANTE T1‐SPACE, the contrast ratios and hyperintense areas were compared between healthy and PD groups, and receiver operating characteristic analyses were performed. We also compared areas under the curve (AUCs) between DANTE T1‐SPACE and the previously reported gradient echo neuromelanin (GRE‐NM) imaging. Each analysis was performed using original images in native space and images transformed into Montreal Neurological Institute space. Values of *P* < 0.05 were considered significant.

**Results:**

DANTE T1‐SPACE showed significantly higher contrast ratios and larger hyperintense areas than T1‐SPACE. On DANTE T1‐SPACE, healthy controls showed significantly higher contrast ratios and larger hyperintense areas than patients with PD. Hyperintense areas in native space analysis achieved the best AUC (0.94). DANTE T1‐SPACE showed AUCs as high as those of GRE‐NM.

**Conclusions:**

DANTE T1‐SPACE successfully visualized neuromelanin of the SNpc and showed potential for evaluating PD. © 2020 The Authors. *Movement Disorders* published by Wiley Periodicals LLC on behalf of International Parkinson and Movement Disorder Society

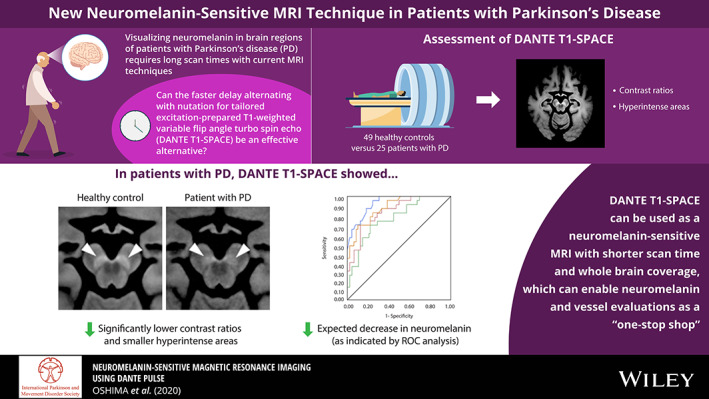

## Graphical abstract

April Infographic: Neuromelanin‐Sensitive Magnetic Resonance Imaging Using DANTE Pulse

Parkinson's disease (PD) is a neurodegenerative disorder involving the progressive loss of dopaminergic neurons of the substantia nigra pars compacta (SNpc); these neurons contain a pigment called neuromelanin.^1^, ^2^ Neuromelanin is a strong chelator of heavy metals, particularly iron, and plays an important role in protection from toxicity caused by free iron.^3^, ^4^ The presymptomatic phase of PD often spans more than 20 years, and symptoms of PD are considered to appear when 50% to 60% of dopaminergic neurons degenerate.^5^, ^6^ Various two‐ and three‐dimensional neuromelanin‐sensitive magnetic resonance imaging (NM‐MRI) methods have been investigated to improve the early diagnosis of PD by exhibiting decreased signal intensity in the SNpc of patients with PD.^7^, ^8^, ^9^, ^10^, ^11^, ^12^, ^13^, ^14^, ^15^, ^16^, ^17^, ^18^, ^19^, ^20^ Magnetization transfer (MT) effects and T1‐shortening effects are considered to contribute strongly to the contrast of this imaging.^7^, ^8^, ^9^, ^10^, ^11^, ^12^, ^13^, ^14^, ^15^, ^17^, ^18^, ^19^, ^21^ Previous studies have shown more prominent reduction in signal intensity in the SNpc as PD advances.^7^, ^17^ Another study found that NM‐MRI reflects the function of SN dopamine neurons.^13^ However, these imaging techniques require relatively long scan times (8–12 minutes) despite limited slice coverage, causing difficulty in obtaining sufficient image quality from patients with tremor or involuntary movements.^7‐10,12‐15,17‐19^

Delay alternating with nutation for tailored excitation (DANTE) pulse has been used for high‐resolution nuclear magnetic resonance spectroscopy, cardiac tagging,^22^ and arterial spin labeling.^23^ DANTE pulse has recently been used for black‐blood imaging in combination with T1‐weighted three‐dimensional turbo spin echo sequence with variable flip angle (T1‐SPACE) in clinical practice to evaluate intracranial and cervical atherosclerotic plaques.^24^, ^25^, ^26^ Three‐dimensional turbo spin echo has a black‐blood effect due to phase dispersion of intravascular signals by the variable flip angle nonselective refocus pulse,^27^, ^28^ and additive DANTE pulse provides a more efficient signal decrease for moving spin such as from intravascular and cerebrospinal fluid.^29^, ^30^

DANTE consists of short, rectangular pulses applied in wideband and provides an MT effect in off‐resonance spectra.^22^ Due to the MT effect of DANTE pulse, we hypothesized that black‐blood DANTE T1‐SPACE for plaque evaluation can also be used as NM‐MRI with shorter scan time for the whole brain than conventional neuromelanin imaging. No previous studies appear to have investigated the effect of DANTE pulse on neuromelanin visualization. The aims of this study were thus to (1) compare the visualization of neuromelanin in the SNpc between DANTE T1‐SPACE and T1‐SPACE without DANTE pulse; (2) assess the ability of DANTE T1‐SPACE to differentiate between healthy and PD groups; and (3) compare the diagnostic ability between DANTE T1‐SPACE and previously reported neuromelanin imaging.^20^

## Patients and Methods

### Study Participants

This study was approved by the institutional review board, and all participants provided written consent. We enrolled 49 healthy controls and 25 patients with PD (Supplementary Material S1). All participants fulfilled the Movement Disorder Society PD Criteria for the diagnosis of PD.^31^ Hoehn and Yahr Scale and Unified Parkinson's Disease Rating Scale (UPDRS), Part III, scores of patients with PD were assessed for both “on” and “off” medication statuses. Imaging was performed for all patients in an “on” medication status. Data were obtained from January 2018 to February 2019. Healthy controls underwent MRI including, DANTE T1‐SPACE, T1‐SPACE, and the previously reported three‐dimensional gradient echo neuromelanin imaging with MT pulse (GRE‐NM),^20^ and patients with PD underwent DANTE T1‐SPACE and GRE‐NM. GRE‐NM images were not available for 2 healthy controls and 3 patients due to technical and/or subject factors. No participant was excluded due to unacceptable image quality.

### Image Acquisition

DANTE T1‐SPACE, T1‐SPACE, and GRE‐NM were performed using a 3‐T MR scanner (MAGNETOM Skyra; Siemens Healthineers, Erlangen, Germany) with a 32‐channel head coil. The imaging parameters for T1‐SPACE were as follows: sagittal acquisition; repetition time/echo time, 1000 ms/11 ms; variable flip angle; echo train length, 60; field of view, 180 × 180 mm; resolution, 0.56 × 0.56 mm; slice thickness, 0.56 mm (isotropic voxel of 0.56 mm); number of slices, 256; controlled aliasing in parallel imaging results in higher acceleration (CAIPIRINHA) acceleration factor, 4; fat suppression. DANTE T1‐SPACE is a prototype sequence for which the DANTE preparation module was added during the acquisition of T1‐SPACE. The parameters for the DANTE pulse were as follows: flip angle, 10°; radiofrequency pulse duration, 0.08 ms; number of pulses, 148; total pulse duration, 167.24 ms; spoiler gradient area, 18 mT/m × ms; and whole‐brain coverage. Acquisition time was 5 minutes 44 seconds for both T1‐SPACE and DANTE T1‐SPACE. The imaging parameters for GRE‐NM were as follows: oblique‐coronal acquisition; repetition time/echo time, 35 ms/2.55 ms; flip angle, 20°; field of view, 178 × 178 mm; resolution, 0.70 × 0.70 mm; slice thickness, 0.70 mm (isotropic voxel of 0.70 mm); number of slices, 60; coverage, 42 mm; and acquisition time, 8 minutes 57 seconds.

### Post‐Imaging Procedure

DANTE T1‐SPACE and GRE‐NM images from each healthy control were co‐registered to T1‐SPACE images of the same control using *SPM12* software (Wellcome Department of Imaging Neuroscience, University College London, United Kingdom) implemented in MATLAB 2014b (Mathworks, Natick, MA). Axially reconstructed images with 0.56‐mm thickness were used for “native space analysis.” A diffeomorphic anatomical registration through exponentiated Lie algebra (DARTEL) template was generated from the image data set of T1‐SPACE. DANTE T1‐SPACE, T1‐SPACE, and GRE‐NM images of healthy controls were warped into Montreal Neurological Institute (MNI) space using the DARTEL template with a smoothing Gaussian filter (full width at half maximum, 0.56 × 0.56 × 0.56 mm), creating spatially normalized DANTE T1‐SPACE, T1‐SPACE, and GRE‐NM. These images were used for “MNI space analysis.” DANTE T1‐SPACE and GRE‐NM for each patient with PD were transferred to MNI space using the same DARTEL template used for healthy controls.

### Image Analysis

All regions of interest (ROIs) and volumes of interest were created using *ImageJ* software (National Institutes of Health, Bethesda, MD) as the consensus decisions of 2 board‐certified radiologists (S.O., 11 years’ experience in neuroradiology, and Y.F., 22 years’ experience in neuroradiology). We excluded pixels with quite low signal intensity from the analysis, as these were considered to represent perivascular space dilations.

### Contrast Ratio Analysis

#### Analysis in Native Space

We created ROI sets of the SNpc and decussation of the superior cerebellar peduncles (SCP) on 5 consecutive slices of images from each participant (Supplementary Material S2). We calculated the contrast ratio as follows: contrast ratio = (SI_SNpc_ – SI_SCP_)/SI_SCP_, where SI_SNpc_ and SI_SCP_ represent the mean signal intensity of the SNpc and SCP, respectively.

#### Analysis in MNI Space

ROIs of the SNpc and SCP were created manually on 5 consecutive slices of spatially normalized averaged images. This ROI set was applied to images from each participant in MNI space. The contrast ratios of the SNpc to the SCP were calculated in the same way as for native space analysis.

### Hyperintense Area Analysis

#### Analysis in Native Space

Following a previously reported threshold signal intensity method,^9^, ^17^ in which the volumes with signal intensity higher than the threshold matched the expected neuromelanin volume, we calculated a threshold signal intensity and measured hyperintense areas in the SNpc. First, we created ROIs of the SNpc and background regions (cerebral peduncles and tegmentum) on 5 consecutive slices (Supplementary Material S2). We then determined the threshold signal intensity for each participant as follows: mean + 3 SD (standard deviation) of background signal intensity. The hyperintense SNpc area was defined as the number of pixels in SNpc ROIs, with signal intensity values higher than the threshold.^9^, ^17^

#### Analysis in MNI Space

ROIs of the SNpc and background region were created manually on 5 consecutive slices of spatially normalized averaged images and were applied to images from each participant in MNI space. We measured the hyperintense SNpc areas in the same way as explained for native space analysis.

### Statistical Analysis

We used *JMP Pro* software, version 14.0 (SAS Institute, Cary, NC). Values of *P* < 0.05 were considered significant. Age and sex distributions were compared between healthy controls and patients with PD using *t* test and χ^2^ test, respectively.

#### Analysis 1: DANTE Effect for SNpc Visualization

We used the Wilcoxon signed‐rank test to assess the differences in contrast ratios and hyperintense areas between DANTE T1‐SPACE and T1‐SPACE images in healthy controls. In addition, we created a DANTE pulse effect map for each healthy control to evaluate the effect of DANTE pulse as follows: (T1‐SPACE – DANTE T1‐SPACE)/T1‐SPACE (%). Each DANTE pulse effect map was also transferred to MNI space using the same DARTEL template. Average spatially normalized DANTE pulse effect maps were created.

#### Analysis 2: Diagnostic Ability of DANTE T1‐SPACE

We evaluated the difference in contrast ratios and hyperintense areas between the healthy and PD groups on DANTE T1‐SPACE images using the Mann‐Whitney *U* test. We also performed receiver operating characteristic curve (ROC) analyses. Areas under the curve (AUCs) and optimal cutoff, sensitivity, and specificity were calculated. We also performed analyses using sex‐matched controls.

In addition, voxel‐based analysis was performed using *SPM12* to detect differences in DANTE T1‐SPACE images between the healthy and PD groups. The mask of the SNpc^32^ was applied to all data to restrict the analysis to the SNpc area, and age and sex were included as covariates, because neuromelanin accumulates in the SN with age.^33^ A 2‐sample *t* test was performed. Values of *P <* 0.001 were considered statistically significant.

We also assessed Pearson's correlation coefficients between UPDRS, Part III, scores (off‐ and on‐medication statuses) and contrast ratios or hyperintense areas.

#### Analysis 3: Comparison of Diagnostic Ability Between DANTE T1‐SPACE and GRE‐NM

We evaluated the differences in contrast ratios and hyperintense areas between the healthy and PD groups in GRE‐NM images using the Mann‐Whitney *U* test. We performed ROC analyses and evaluated the differences in AUCs between DANTE T1‐SPACE and GRE‐NM using the DeLong test.^34^ We also assessed Spearman's rank correlation coefficients between DANTE T1‐SPACE and GRE‐NM for contrast ratios and hyperintense areas. We calculated Dice similarity coefficients of hyperintense areas between DANTE T1‐SPACE and GRE‐NM. To further understand the relationships between DANTE T1‐SPACE and GRE‐NM, we performed supplementary analyses of hyperintense areas using the threshold of mean + 1.5 SD of the background signal intensity.^35^

## Results

### Study Cohort Demographics

A total of 74 participants were included in the analysis, comprising 49 healthy controls (mean [±SD] age, 58.0 ± 13.9 years; 30 women) and 25 patients with PD (mean age, 60.3 ± 7.8 years; 14 men). Participant characteristics are provided in Table 1. Hoehn and Yahr Scale and UPDRS, Part III, scores are shown for patients with PD. In the sex‐matched analysis, we evaluated 25 healthy controls (mean age, 66.1 ± 6.8 years; 14 men [67.4 ± 7.7 years] and 11 women [64.4 ± 4.9 years]) and 25 patients with PD (14 men [54.1 ± 5.7 years] and 11 women [65.6 ± 6.9 years]).

**TABLE 1:** Characteristics of healthy controls and patients with PD

Characteristics		Healthy controls	PD patients	*P‐*value
Number		49	25	
Age (yr)	Mean ± standard deviation	58.0 ± 13.9	60.3 ± 7.8	0.45
Sex	Number male/female	19/30	14/11	0.16
HY stage (medication on)	Median (range)	–	2 (1–3)	
HY stage (medication off)	Median (range)	–	2 (1–5)	
UPDRS, Part III (medication on)	Median (range)	–	19 (10–34)	
UPDRS, Part III (medication off)	Median (range)	–	42 (18–63)	
Disease duration (yr)	Median (range)	–	8 (2–18)	

Abbreviations: PD, Parkinson's disease; HY, Hoehn and Yahr; UPDRS, Unified Parkinson's Disease Rating Scale.

#### Analysis 1: DANTE Effect for SNpc Visualization

**FIG. 1:**
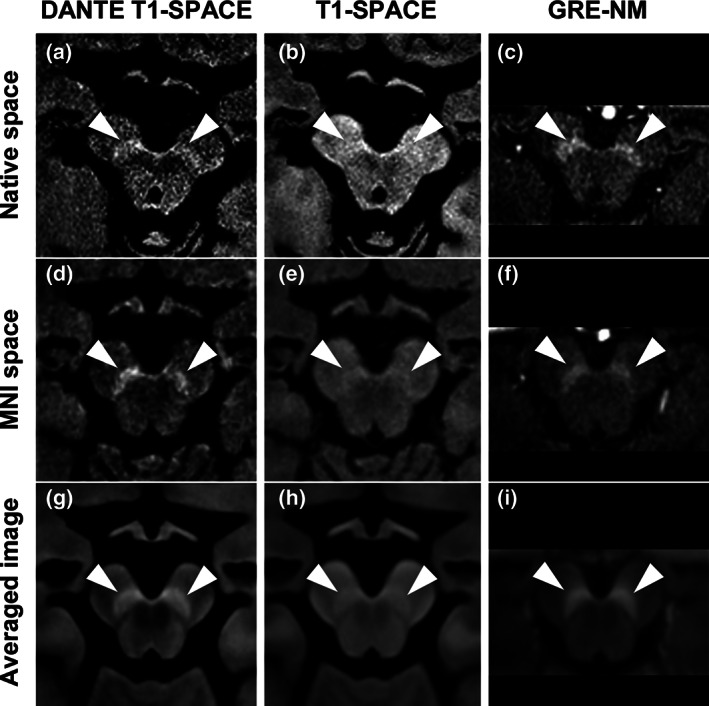
DANTE T1‐SPACE, T1‐SPACE, and GRE‐NM for a healthy participant in native space (upper row) and Montreal Neurological Institute (MNI) space (middle row). Lower row shows averaged images of all healthy participants in MNI space. Arrowheads indicate the substantia nigra pars compacta.

Representative images from DANTE T1‐SPACE, T1‐SPACE, and GRE‐NM imaging of a 74‐year‐old male participant are shown in Figure 1. Figure 1d‐f shows images after transformation to the MNI space. Averaged images for DANTE T1‐SPACE, T1‐SPACE, and GRE‐NM in MNI space of all healthy participants are presented in (Fig. 1g‐i) to show the differences in these imaging methods more clearly. DANTE T1‐SPACE was able to delineate the SNpc much better than T1‐SPACE. A DANTE pulse effect map is shown in Supplementary Material S3, demonstrating that DANTE pulse increases the signal intensity of the SNpc and suppresses the signal intensity of the midbrain background area, leading to delineation of the SNpc as a high‐intensity area.

Contrast ratios of SNpc in DANTE T1‐SPACE were significantly higher than those in T1‐SPACE without DANTE pulse in native space (mean ± SD: DANTE T1‐SPACE, 23.64 ± 4.51; T1‐SPACE, 14.71 ± 3.68; *P* < 0.001) and in MNI space analyses (DANTE T1‐SPACE, 25.34 ± 4.11; T1‐SPACE, 15.98 ± 3.50; *P* < 0.001) (Supplementary Material S4). Hyperintense areas were significantly larger for DANTE T1‐SPACE than for T1‐SPACE in native space (DANTE T1‐SPACE, 16.58 ± 6.33; T1‐SPACE, 7.86 ± 3.87; *P* < 0.001) and in MNI space analyses (DANTE T1‐SPACE, 65.87 ± 26.88; T1‐SPACE, 10.82 ± 10.52; *P* < 0.001) (Supplementary Material S4).

#### Analysis 2: Diagnostic Ability of DANTE T1‐SPACE

**FIG. 2:**
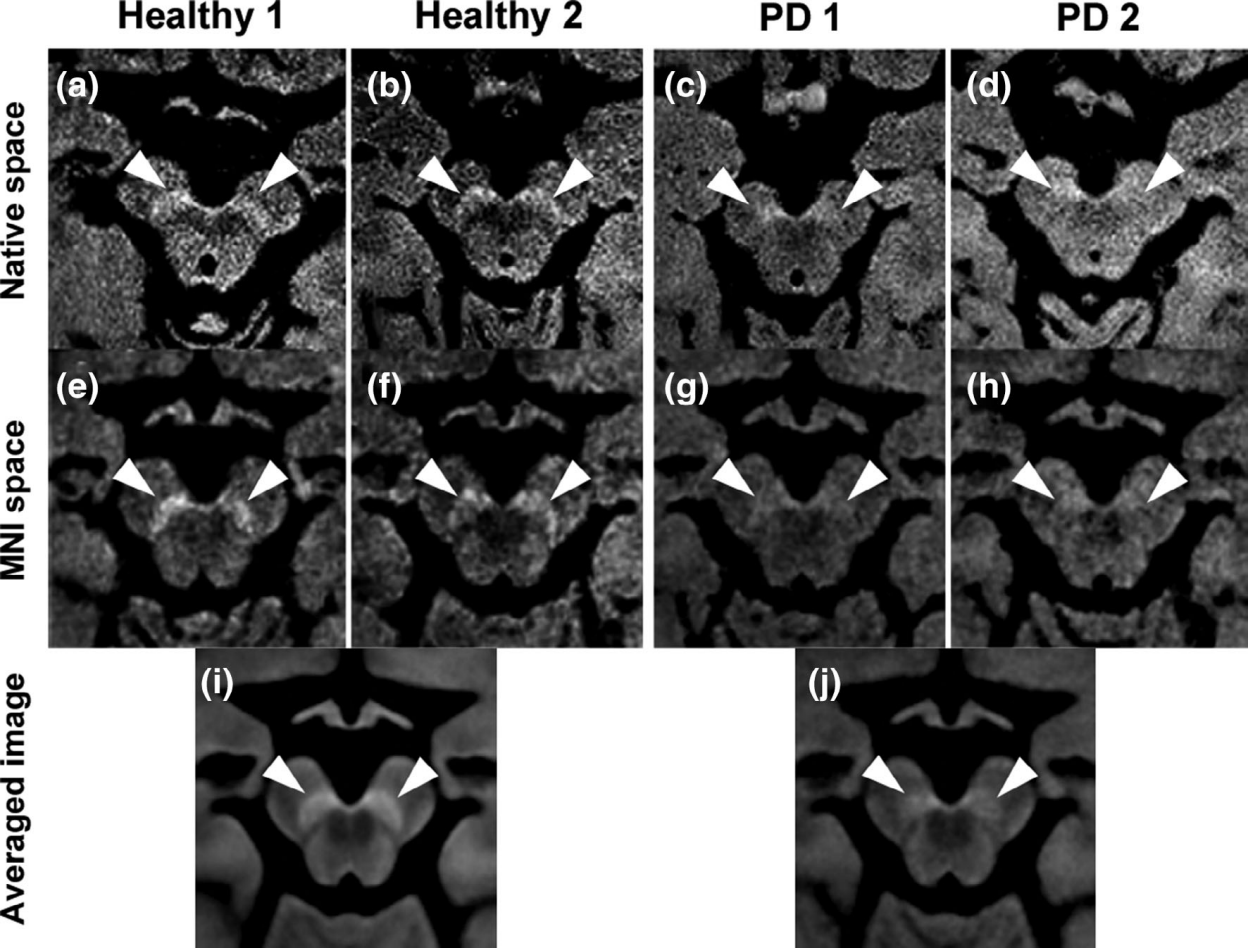
DANTE T1‐SPACE images of (**a, b, e, f**) healthy controls and (**c, d, g, h**) patients with Parkinson's disease in native space (upper) and Montreal Neurological Institute (MNI) space (middle). Lower row shows averaged images in MNI space. Arrowheads indicate substantia nigra pars compacta.

Representative DANTE T1‐SPACE images of healthy controls and patients with PD are shown in Figure 2 (a and e: a 74‐year‐old male control; b and f: a 65‐year‐old female control; c and g: a 60‐year‐old female patient; d and h: a 59‐year‐old female patient). Figure 2e‐h shows images after transformation into the MNI space. Averaged images of DANTE T1‐SPACE in MNI space for healthy controls and patients with PD are presented in Figure 2i,j. SNpc was more clearly delineated in healthy controls than in patients with PD.

**FIG. 3:**
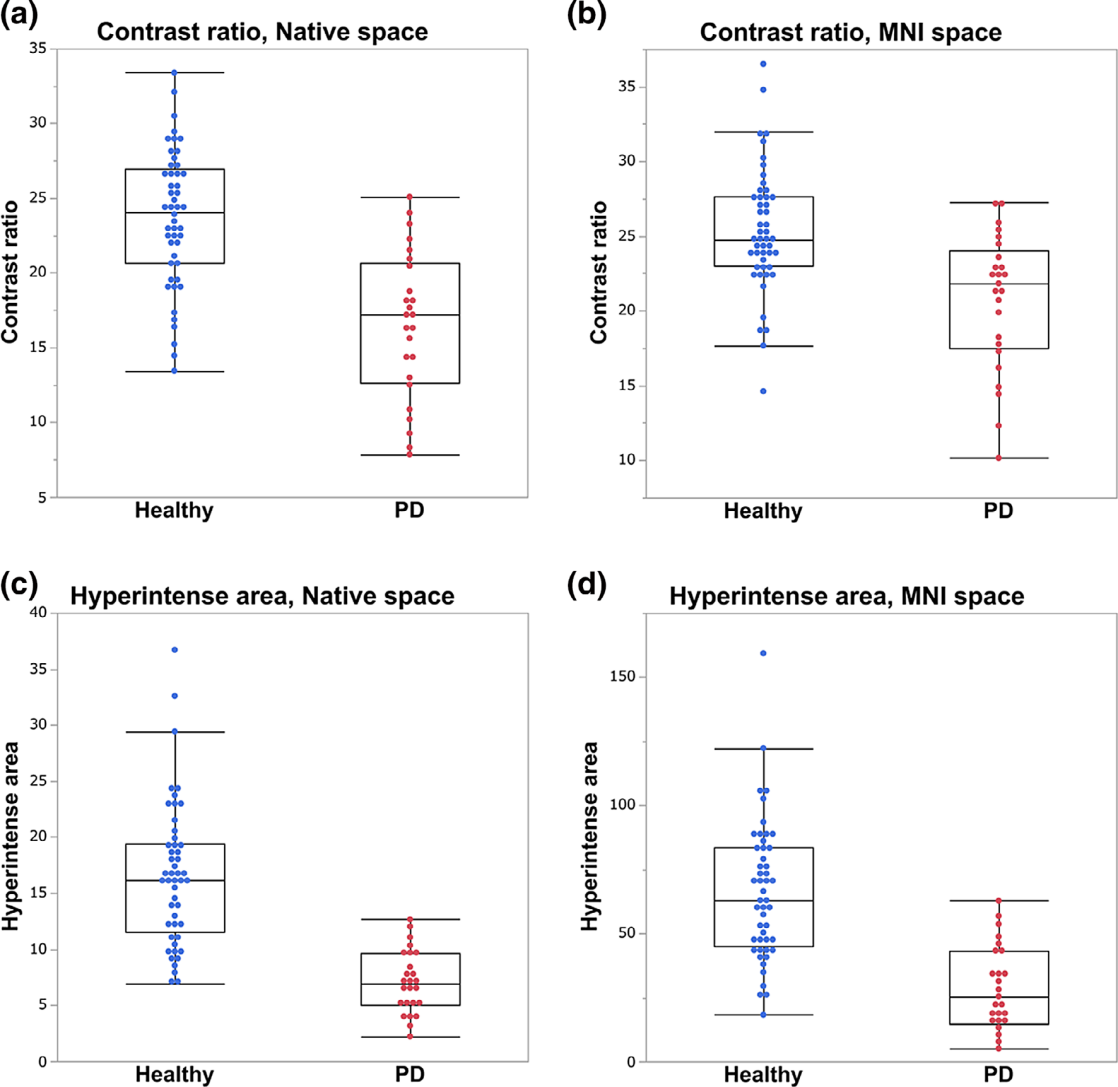
Box‐and‐whisker and scatter plots of healthy controls versus patients with Parkinson's disease for (**a, b**) contrast ratio analysis and (**c, d**) hyperintense area analysis using DANTE T1‐SPACE images in (**a, c**) native space and (**b, d**) Montreal Neurological Institute space. [Color figure can be viewed at wileyonlinelibrary.com]

**FIG. 4:**
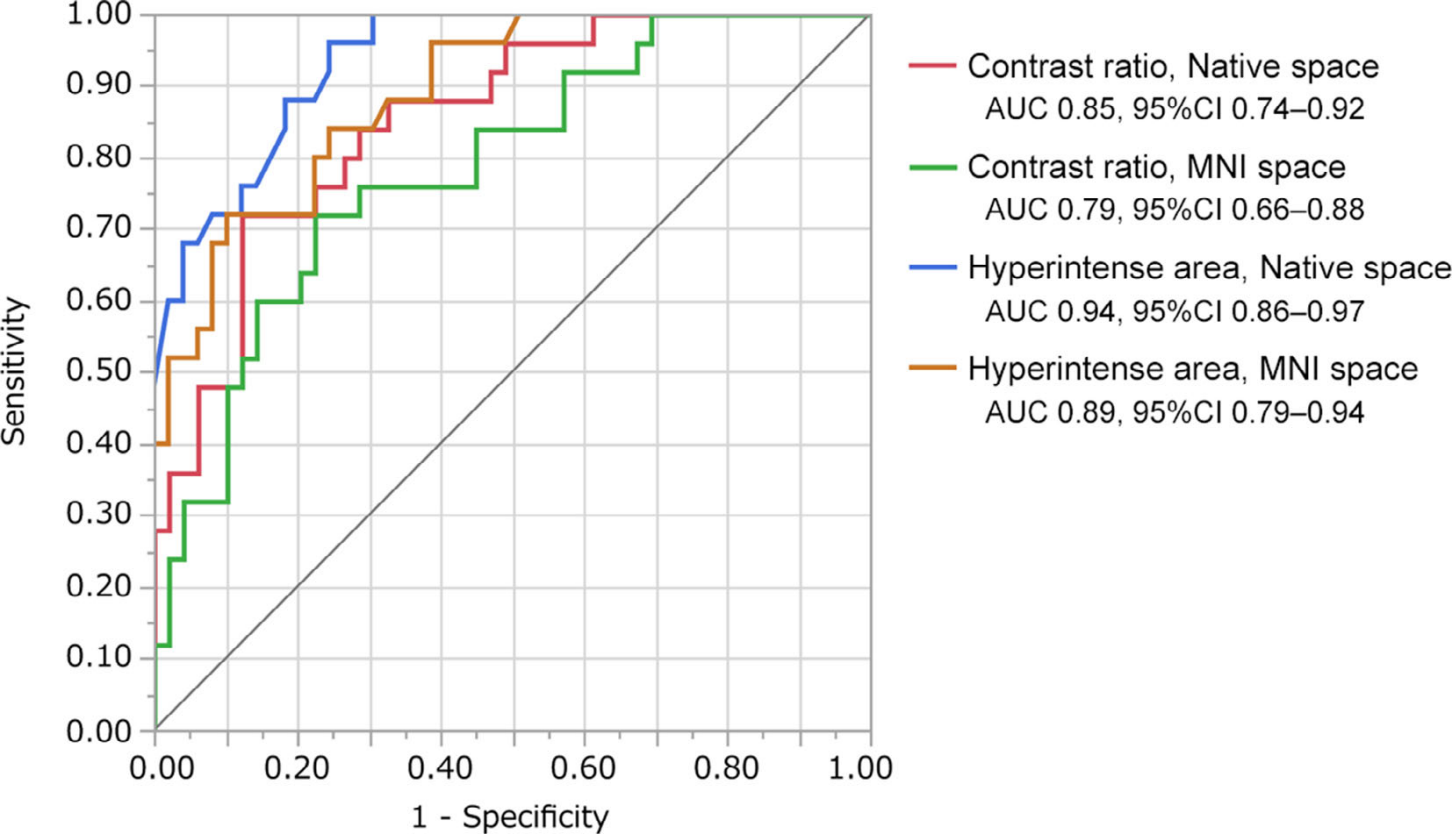
Receiver operating characteristic curves of contrast ratios and hyperintense areas in native space and in Montreal Neurological Institute space analyses for diagnosis of Parkinson's disease using DANTE T1‐SPACE images. [Color figure can be viewed at wileyonlinelibrary.com]

Contrast ratios of SNpc were significantly higher for healthy controls than for patients with PD in native space (healthy, 23.64 ± 4.51; PD, 16.53 ± 4.85; *P* < 0.001) and in MNI space analyses (healthy, 25.34 ± 4.11; PD, 20.68 ± 4.42; *P* < 0.001) (Fig. 3, Supplementary Material S5). Hyperintense areas were significantly larger for healthy controls than for patients with PD in native space (healthy, 16.58 ± 6.33; PD, 7.10 ± 2.73; *P* < 0.001) and in MNI space analyses (healthy, 65.87 ± 26.88; PD, 28.40 ± 16.36; *P* < 0.001) (Fig. 3, Supplementary Material S5). AUCs were 0.85 (95% confidence interval [CI]: 0.74–0.92) (contrast ratios in native space), 0.79 (95% CI: 0.66–0.88) (contrast ratios in MNI space), 0.94 (95% CI: 0.86–0.97) (hyperintense areas in native space), and 0.89 (95% CI: 0.79–0.94) (hyperintense areas in MNI space) (Fig. 4, Supplementary Material S5). In the sex‐matched analysis, we also found significant differences between healthy and PD groups. AUCs were 0.83 (contrast ratios in native space), 0.72 (contrast ratios in MNI space), 0.93 (hyperintense areas in native space), and 0.92 (hyperintense areas in MNI space) (Supplementary Materials S6 and S7). The results of group comparisons between healthy controls and patients with PD using DANTE T1‐SPACE after adjusting for age and sex are shown in Supplementary Material S8. Voxels with significantly higher signals in the healthy group compared to the PD group were observed in the SNpc. We found no significant correlation between UPDRS, Part III, scores and contrast ratios or hyperintense areas.

#### Analysis 3: Comparison of Diagnostic Ability Between DANTE T1‐SPACE and GRE‐NM

The results of contrast ratios and hyperintense areas of healthy and PD groups using DANTE T1‐SPACE and GRE‐NM are shown in Supplementary Material S9. In ROC analyses for diagnosing PD, AUCs for DANTE T1‐SPACE and GRE‐NM were 0.85 (95% CI: 0.74–0.92) and 0.84 (95% CI: 0.71–0.91) for contrast ratios in native space, 0.79 (95% CI: 0.66–0.88) and 0.80 (95% CI: 0.67–0.88) for contrast ratios in MNI space, 0.94 (95% CI: 0.87–0.98) and 0.92 (95% CI: 0.81–0.96) for hyperintense areas in native space, and 0.89 (95% CI: 0.79–0.95) and 0.83 (95% CI: 0.70–0.91) for hyperintense areas in MNI space, respectively (Supplementary Material S10). No significant differences in AUCs were identified between DANTE T1‐SPACE and GRE‐NM in any of those 4 analyses. Correlations between DANTE T1‐SPACE and GRE‐NM are shown in Supplementary Material S11. Weak correlations were apparent in healthy controls and the total cohort, but no significant correlations were evident in patients with PD except for hyperintense areas in native space. Dice similarity coefficients between DANTE T1‐SPACE and GRE‐NM were 0.45 ± 0.11 (mean ± SD) in healthy controls, 0.20 ± 0.12 in patients with PD, and 0.37 ± 0.16 in the total cohort. The results of analyses using the threshold of mean + 1.5 SD are shown in Supplementary Material S12.

## Discussion

This study demonstrated that DANTE T1‐SPACE not only is a high‐resolution black‐blood imaging sequence but also has the potential as NM‐MRI. DANTE T1‐SPACE of healthy participants showed much better visualization of neuromelanin in the SNpc than T1‐SPACE without DANTE pulse. The contrast ratios were significantly higher and the hyperintense areas were significantly larger for DANTE T1‐SPACE than for T1‐SPACE in both native space and MNI space analyses. Although the underlying contrast mechanisms remain unclear on NM‐MRI, MT effects are considered to contribute strongly to neuromelanin‐sensitive contrast.^7^, ^8^, ^9^, ^10^, ^11^, ^12^, ^13^, ^14^, ^15^, ^17^, ^18^, ^19^, ^21^ The paramagnetic properties of metal‐bound neuromelanin can cause a T1‐shortening effect,^8^, ^9^, ^10^, ^11^, ^12^, ^16^, ^17^, ^18^, ^36^ but the high signal intensity of neuromelanin on turbo spin echo sequences may also be due to incidental MT effects associated with multi‐slice acquisition.^37^ Our study showed that SNpc could be visualized more clearly by adding the DANTE pulse to T1‐SPACE. DANTE pulse comprises short, rectangular pulses applied in wideband and provides MT effects in off‐resonance spectra, which are considered to contribute to the neuromelanin contrast.^22^

As for diagnostic performance, DANTE T1‐SPACE successfully differentiated patients with PD from healthy controls with good AUCs. Both native‐space and MNI‐space analyses indicated that contrast ratios and hyperintense areas were significantly decreased in patients with PD. This is consistent with a previous study showing reduced contrast in the SN among patients with PD on NM‐MRI. The best AUC was observed for hyperintense area analysis in native space (AUC = 0.94). Further studies with more participants are expected to assess the best method for determining thresholds for the diagnosis of PD with DANTE T1‐SPACE.

We also performed a comparative study of diagnostic ability between DANTE T1‐SPACE and GRE‐NM. The AUCs of DANTE T1‐SPACE for the diagnosis of PD were as high as GRE‐NM, and no significant difference was found between these two imaging methods. Our results suggest that DANTE T1‐SPACE has the potential for diagnosing PD.

Whether contrast ratios or hyperintense areas on NM‐MRI correlate with UPDRS, Part III, scores remains controversial.^9^, ^35^ The lack of evidence for a correlation in our study may be due to the high interindividual variability of SN volumes, as described in postmortem volumetric studies,^38^ and discrepancies between motor symptoms and neuromelanin loss. In addition, the sample size in our study was not large enough to identify correlations between UPDRS, Part III, scores and contrast ratios or hyperintense areas. However, one of the clinical needs for PD biomarkers is early diagnosis within the prodromal period. About half of the dopamine neurons usually degenerated by the time of clinical diagnosis,^5^, ^6^ making DANTE T1‐SPACE a promising candidate for early detection of PD.

DANTE T1‐SPACE is a prototype black‐blood imaging method and has proven useful for intra‐ and extracranial plaque evaluations.^24^, ^25^, ^26^, ^27^, ^28^, ^29^ Vascular assessment is important for ruling out vascular parkinsonism or vascular dementia, which are sometimes difficult to differentiate from PD. Our study suggests that DANTE T1‐SPACE may enable the evaluation of vessels and neuromelanin quantification as a “one‐stop shop.” Various NM‐MRI methods have been investigated but require relatively long scan times and offer limited slice coverage.^7^, ^8^, ^9^, ^10^, ^11^, ^12^, ^13^, ^14^, ^15^, ^16^, ^17^, ^18^, ^19^, ^20^ One of the advantages of the DANTE T1‐SPACE used in this study was the relatively short scan time (5 minutes 44 seconds) for whole‐brain coverage, due to the acceleration factor of 4 in CAIPIRINHA. This is highly advantageous to examine patients with PD, who often experience difficulty in maintaining a steady head position. In addition, the high resolution of this imaging method reduces the partial volume effect to evaluate small structures such as the SN, which is less than 15 mm in height.^32^ Whole‐brain coverage is also advantageous. This is because atlas‐based segmentation approaches have recently proven effective for evaluating the SNpc and diagnosing PD,^3^, ^4^ and whole‐brain scans make such approaches easier than reduced field‐of‐view acquisitions.^14^

The first limitation of this study was the small number of participants and the relatively advanced stage of PD. Further studies with a larger number of participants are required. Second, variability could have existed between subjects regarding the slice selection and ROI placement in the analysis of images in native space because of the differences between subjects and the difficulty of defining borders of the SN in patients with PD due to the loss of neuromelanin‐containing neurons. To decrease such bias, we also performed analyses using the same ROIs on images transformed into MNI space. Third, we identified weak or no correlations of contrast ratios or hyperintense areas between DANTE T1‐SPACE and GRE‐NM. Further, Dice similarity coefficients of hyperintense areas were low. This was attributed to the following reasons: different types of MT effects (DANTE pulse or ordinal MT pulse), minor misregistration due to different coverages (whole brain or limited to the brainstem), and the limited number of subjects. The relatively small size of hyperintense areas may have reduced overlap between DANTE T1‐SPACE and GRE‐NM, resulting in low Dice similarity coefficients. Fourth, the implementation of the DANTE pulse in the present study was optimized for the black‐blood effect and not necessarily optimal for neuromelanin imaging. We are therefore currently investigating the effects of altering the duty cycle, pulse separation, and pulse amplitude, with a view to further reducing power deposition while maintaining the black‐blood effect. Finally, we did not evaluate age‐related changes of NM‐MRI in this study, because we did not include enough young subjects. In a previous study,^33^ NM‐MRI indicated an inverted U‐shaped association of SNpc signal intensity in cohorts from childhood to old age. Further studies are required to evaluate the change in neuromelanin amount with age using a larger cohort of subjects with a more proportional age distribution.

In conclusion, DANTE T1‐SPACE can visualize neuromelanin in the SNpc and represents a potential tool for diagnosing PD.

## Author Roles

(1) Research project: A. Conception, B. Organization, C. Execution; (2) Statistical analysis: A. Design, B. Execution, C. Review and critique; (3) Manuscript preparation: A. Writing of the first draft, B. Review and critique.

S.O.: 1A, 1B, 1C, 2A, 2B, 2C, 3A, 3B

Y.F.: 1A, 1B, 1C, 2A, 2B, 2C, 3A, 3B

T.O.: 1A, 1B, 1C, 2A, 2C, 3B

S.N.: 1A, 1B, 1C, 2A, 2C, 3B

Y.Y.: 1A, 1B, 1C, 2A, 2C, 3B

A.S.: 1A, 1B, 1C, 2C, 3B

J.G.: 1A, 1B, 1C, 2C, 3B

S.A.: 1A, 1B, 1C, 2C, 3B

N.S.: 1A, 1B, 1C, 2C, 3B

R.T.: 1A, 1B, 1C, 2C, 3B

Y.N.: 1A, 1B, 1C, 2C, 3B

## Full financial disclosures for the previous 12 months

S.O. has nothing to disclose. Y.F. has received JSPS KAKENHI Grant Number JP18K07711 and has a patent JP 2019‐063535 A. T.O. has received JSPS KAKENHI Grant Number JP18K18453, grant support from Siemens Healthcare Japan K.K., and honoraria from Guerbet Japan K.K. S.N. has received JSPS KAKENHI Grant Number JP19K17266. Y.Y. has nothing to disclose. A.S. has received the internal grant of Kyoto University, Start Up. J.G. and S.A. are employees of Siemens Healthineers. N.S. has received AMED Grant Number JP18dm0307003 and grant support from Otsuka Pharmaceutical and Kyowa Kirin. R.T. has received grant support from Takeda Pharmaceutical, Sumitomo Dainippon Pharma, Eisai, and Otsuka Pharmaceutical; honoraria from Takeda Pharmaceutical, Sumitomo Dainippon Pharma, AbbVie, Otsuka Pharmaceutical, Eisai, FP Pharmaceutical, and Kyowa Kirin; and consulting fee from Kan Institute. Y.N. has received JSPS KAKENHI Grant Number JP20K07657 and grant support from Shimadzu.

## Supporting information


**Supplementary Material S1.** Flowchart of participant enrollment.
**Supplementary Material S2.** ROIs for contrast ratio analysis and hyperintense area analysis in (**A**, **B**) native space and (**C**, **D**) MNI space. (**B**, **D**) Binary images represent pixels with signals above the threshold. Arrowheads, SNpc; arrows, SCP.
**Supplementary Material S3.** Averaged images of DANTE T1‐SPACE (left) and T1‐SPACE (middle). The right column shows DANTE pulse effect maps, which were created as (T1‐SPACE – DANTE T1‐SPACE)/T1‐SPACE (%). The scale bar is for the DANTE pulse effect map.
**Supplementary Material S4.** Box‐and‐whisker and scatter plots of DANTE T1‐SPACE versus T1‐SPACE of healthy participants for (**A**, **B**) contrast ratio analysis and (**C**, **D**) hyperintense area analysis in (**A**, **C**) native space and (**B**, **D**) MNI space.
**Supplementary Material S5.** Results of analysis 2 (healthy controls vs. PD patients in DANTE T1‐SPACE). (**A**) Contrast ratios and hyperintense areas of healthy controls and PD patients; (**B**) ROC analysis for differentiating participants with PD from healthy participants using DANTE T1‐SPACE.
**Supplementary Material S6.** Results of the sex‐matched analysis. (**A**) Contrast ratios and hyperintense areas on DANTE T1‐SPACE for healthy controls and patients with PD; (**B**) ROC analysis for differentiating participants with PD from healthy participants using DANTE T1‐SPACE.
**Supplementary Material S7.** ROC curves for diagnosing PD using DANTE T1‐SPACE images in the sex‐matched analysis.
**Supplementary Material S8.** Results of voxel‐based analysis on DANTE T1‐SPACE between healthy and PD groups. Color indicates voxels where signal intensity was statistically higher in the healthy group compared to PD groups after age‐ and sex adjustment (*P* < 0.001). Color bar indicates T‐values.
**Supplementary Material S9.** Contrast ratios and hyperintense areas of healthy controls and PD patients in analysis 3 (comparison study between DANTE T1‐SPACE and GRE‐NM).
**Supplementary Material S10.** ROC curves of DANTE T1‐SPACE and GRE‐NM for diagnosis of PD. (**A**) Contrast ratios in native space analysis; (**B**) contrast ratios in MNI space analysis; (**C**) hyperintense areas in native space analysis; (**D**) hyperintense areas in MNI space analysis.
**Supplementary Material S11.** Correlation coefficients (R^2^) of contrast ratios and hyperintense areas for comparisons between DANTE T1‐SPACE and GRE‐NM images.
**Supplementary Material S12.** Results of analyses using threshold of mean + 1.5 standard deviations. (**A**) Hyperintense areas of healthy controls and patients with PD on DANTE T1‐SPACE and GRE‐NM using the threshold of mean + 1.5 standard deviations of background area; (**B**) ROC curves of hyperintense areas using the threshold of mean + 1.5 standard deviations of background area; (**C**) Dice similarity coefficients of hyperintense areas in MNI space between DANTE T1‐SPACE and GRE‐NM.
