# Supplementary material for: Neuromelanin‐Sensitive Magnetic Resonance Imaging Using DANTE Pulse
Source: Mov Disord. 2020 Dec 14;36(4):874–82. doi: 10.1002/mds.28417 (PMC8247273; doi:10.1002/mds.28417)
Supplement: Supplementary file 1 — Supplementary Material S1. Flowchart of participant enrollment. Supplementary Material S2. ROIs for contrast ratio analysis and hyperintense area analysis in (A, B) native space and (C, D) MNI space. (B, D) Binary images represent pixels with signals above the threshold. Arrowheads, SNpc; arrows, SCP. Supplementary Material S3. Averaged images of DANTE T1‐SPACE (left) and T1‐SPACE (middle). The right column shows DANTE pulse effect maps, which were created as (T1‐SPACE – DANTE T1‐SPACE)/T1‐SPACE (%). The scale bar is for the DANTE pulse effect map. Supplementary Material S4. Box‐and‐whisker and scatter plots of DANTE T1‐SPACE versus T1‐SPACE of healthy participants for (A, B) contrast ratio analysis and (C, D) hyperintense area analysis in (A, C) native space and (B, D) MNI space. Supplementary Material S5. Results of analysis 2 (healthy controls vs. PD patients in DANTE T1‐SPACE). (A) Contrast ratios and hyperintense areas of healthy controls and PD patients; (B) ROC analysis for differentiating participants with PD from healthy participants using DANTE T1‐SPACE. Supplementary Material S6. Results of the sex‐matched analysis. (A) Contrast ratios and hyperintense areas on DANTE T1‐SPACE for healthy controls and patients with PD; (B) ROC analysis for differentiating participants with PD from healthy participants using DANTE T1‐SPACE. Supplementary Material S7. ROC curves for diagnosing PD using DANTE T1‐SPACE images in the sex‐matched analysis. Supplementary Material S8. Results of voxel‐based analysis on DANTE T1‐SPACE between healthy and PD groups. Color indicates voxels where signal intensity was statistically higher in the healthy group compared to PD groups after age‐ and sex adjustment (P < 0.001). Color bar indicates T‐values. Supplementary Material S9. Contrast ratios and hyperintense areas of healthy controls and PD patients in analysis 3 (comparison study between DANTE T1‐SPACE and GRE‐NM). Supplementary Material S10. ROC curves of DANTE T1‐SPACE and GRE‐NM [file MDS-36-874-s001.zip › MDS_28417_S11.docx]

**Supplementary Material 11**

**Correlation coefficients (R^2^) of contrast ratios and hyperintense areas for comparisons between DANTE T1-SPACE and GRE-NM images**

|  | | Healthy (n = 47) | PD (n = 22) | All (n = 69) |
| --- | --- | --- | --- | --- |
| Contrast ratio | Native space | 0.21 (*p* = 0.001) | 0.06 (*p* = 0.28) | 0.23 (*p* < 0.001) |
|  | MNI space | 0.31 (*p* < 0.001) | 0.10 (*p* = 0.15) | 0.37 (*p* < 0.001) |
| Hyperintense  area | Native space | 0.11 (*p* = 0.02) | 0.18 (*p* = 0.048) | 0.40 (*p* < 0.001) |
|  | MNI space | 0.12 (*p* = 0.02) | 0.02 (*p* = 0.53) | 0.31 (*p* < 0.001) |

DANTE T1-SPACE = delay alternating with nutation for tailored excitation-prepared T1-weighted variable flip angle turbo spin echo; GRE-NM = three-dimensional gradient echo neuromelanin imaging; PD = Parkinson’s disease; MNI = Montreal Neurological Institute.
